# Giant Self-Kerr Nonlinearity in the Metal Nanoparticles-Graphene Nanodisks-Quantum Dots Hybrid Systems Under Low-Intensity Light Irradiance

**DOI:** 10.3390/nano8070521

**Published:** 2018-07-12

**Authors:** Mariam M. Tohari, Andreas Lyras, Mohamad S. AlSalhi

**Affiliations:** 1 Department of Physics and Astronomy, College of Science, King Saud University, P.O. Box 2454, Riyadh 11451, Saudi Arabia; alyras@ksu.edu.sa (A.L.); malsalhi@ksu.edu.sa (M.S.A.); 2 Department of Physics, College of Science, King Khalid University, P.O. Box 9004, Abha 61421, Saudi Arabia; 3 Research Chair on Laser Diagnosis of Cancers, College of Science, King Saud University, P.O. Box 2454, Riyadh 11451, Saudi Arabia

**Keywords:** self-Kerr nonlinearity, electromagnetically induced transparency, graphene nanodisks, metal nanoparticles, self-assembled quantum dots

## Abstract

Hybrid nanocomposites can provide a promising platform for integrated optics. Optical nonlinearity can significantly widen the range of applications of such structures. In the present paper, a theoretical investigation is carried out by solving the density matrix equations derived for a metal nanoparticles-graphene nanodisks-quantum dots hybrid system interacting with weak probe and strong control fields, in the steady state. We derive analytical expressions for linear and third-order nonlinear susceptibilities of the probe field. A giant self-Kerr nonlinear index of refraction is obtained in the optical region with relatively low light intensity. The optical absorption spectrum of the system demonstrates electromagnetically induced transparency and amplification without population inversion in the linear optical response arising from the negative real part of the polarizabilities for the plasmonic components at the energy of the localized surface plasmon resonance of the graphene nanodisks induced by the probe field. We find that the self-Kerr nonlinear optical properties of the system can be controlled by the geometrical features of the system, the size of metal nanoparticles and the strength of the control field. The controllable self-Kerr nonlinearities of hybrid nanocomposites can be employed in many interesting applications of modern integrated optics devices allowing for high nonlinearity with relatively low light intensity.

## 1. Introduction

Nonlinear optics play an important role in modern photonics enabling various applications including; frequency conversion [1], ultrafast lasers and amplifiers [2,3], ultrafast all-optical switching [4] and nonlinear microscopy [5]. Usually very large field intensities are required to manipulate the optical properties of materials in order to obtain nonlinear effects [6]. However, one way to provide efficient nonlinear devices with low light intensity is to use the strong localization of electromagnetic field in the form of surface plasmon polaritons [7]. Therefore, significant efforts have been devoted to studying both theoretically and experimentally the nonlinearity at the nanoscale using plasmonic structures [8,9,10,11,12].

The metal nanoparticles (MNPs) can enhance the nonlinear optical response due to the large local field enhancement induced near the surface and control the optical properties of quantum emitters near the MNPs [13,14,15]. Moreover, plasmonic excitations can respond within femtoseconds enabling ultrafast processing of optical signal [16], with high sensitivity to the size and the shape of the MNPs as well as the dielectric properties of the metal and surrounding medium [17]. Interestingly, being a semimetal with linear dispersion relation for its high-mobility charge carriers, graphene can enhance light-matter interactions supporting high nonlinearity effects [18,19,20]. Moreover, due to the three dimensional confinement, the extreme localization of plasmonic fields in graphene nanodisks (GNDs) results in many nonlinear effects such as plasmon blockade and solitons [21,22]. The local field induced in composite nanostructures of both noble metals and graphene can lead to increased nonlinearity, either intrinsic nonlinearity, i.e., within the plasmonic structure, or extrinsic nonlinearity, i.e., in the adjacent dielectric medium. However, if both sources of nonlinearity are present, usually the latter tends to be stronger [23,24]. To compensate for the loss in plasmonic structures, gain media such as quantum dots (QDs) are incorporated within the system which in turn demonstrate considerable nonlinearities employed in various applications of optoelectronics devices [25,26].

Another way to induce nonlinear effects with low light intensity, is electromagnetically induced transparency (EIT). EIT employs the atomic coherence induced in multilevel atomic systems to make the absorptive medium transparent to a resonant probe field due to the destructive quantum interference induced between two excitation pathways via a control field [27]. The steep dispersion associated with EIT can reduce significantly the group velocity of propagating light and increase the time of interaction between light and matter resulting in more efficient nonlinear optical effects [28,29]. H. Schmidt and A. Imamoglu have obtained giant Kerr nonlinearities by EIT [30]. Wang et al. found that the Kerr nonlinear index of refraction of a three-level Λ type atomic system is greatly enhanced inside an optical ring cavity near resonance for both probe and control fields [31].

Additionally, the effects of optical transparency can be created and controlled in the presence of plasmonic nanostructures [32,33]. Moreover, the Kerr nonlinearity can be significantly enhanced in hybrid nanocomposites providing a promising platform for integrated optics with potential nonlinear applications [34,35,36]. Therefore, the Kerr nonlinearity has been investigated for different hybrid systems in order to study the role of exciton-plasmon coupling [37,38] and investigate the contribution of both QD and MNP susceptibilities in the total nonlinear susceptibility of the hybrid system [39]. Interestingly, the MNP-GND-QD hybrid systems demonstrate controllable ultrafast energy exchange between plasmons and excitons [40], that can be associated with high Kerr nonlinearity.

**Figure 1:**
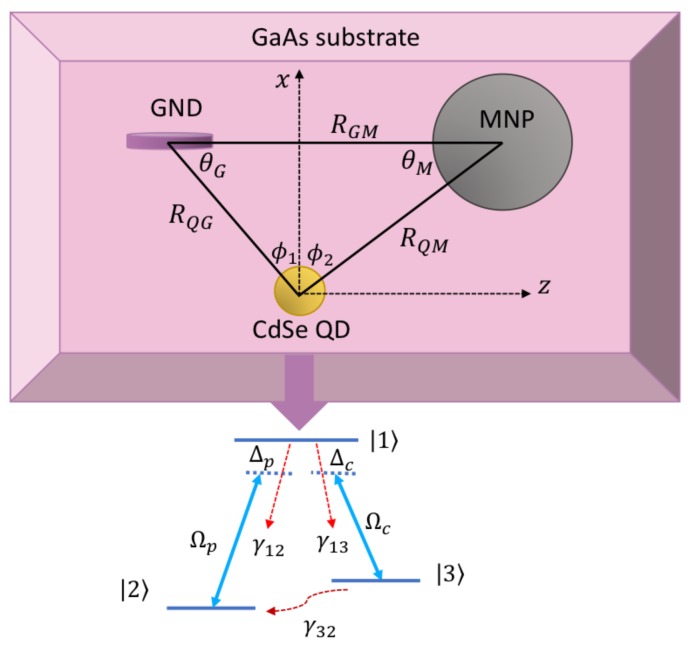
The proposed model of MNP-GND-QD hybrid system deposited on a GaAs substrate.

In the present work we will study the self-Kerr nonlinearity in the MNP-GND-QD hybrid system, depicted in Figure 1, in the optical region of the electromagnetic spectrum. It has been already demonstrated that in this hybrid system the dipole-dipole interaction (DDI) between the components of the hybrid system is enhanced in the optical range of the electromagnetic spectrum [40]. To enhance the nonlinearity of the system, we consider self-assembled QD modeled as three level atomic systems in a Λ configuration shown in Figure 1 that support EIT in the presence of weak probe and strong control fields that induce the optical excitations in the components of the system [41]. The density matrix equations derived within the rotating wave approximation will be solved for the steady state in the weak probe field limit, to obtain linear and third-order nonlinear susceptibilities for the probe field that induces surface plasmon polaritons in the GND, resonant with excitons in the QD. We will study the self-Kerr nonlinearity under various conditions related to the geometry of the system and the strength of the control field.

## 2. Theoretical Formalism

We consider the MNP-GND-QD hybrid system deposited on the GaAs substrate as shown in Figure 1. The QD is assumed to be a three-level system of Λ configuration. Under the assumption that the dipole matrix elements $\mu_{12}$ and $\mu_{13}$ lie along *x* and *z* directions, respectively, and by applying the probe and control fields along x and z directions respectively, the probe field of frequency $\omega_{p}$ and Rabi frequency $\Omega_{p}$ induces the transition |1〉↔|2〉 which is resonant with GND surface plasmons $\hbar \omega_{sp}^{x}$. On the other hand, the control field of $\omega_{c}$ and $\Omega_{c}$ drives the transition |1〉↔|3〉 and does not couple to surface plasmons since it is far detuned from $\hbar \omega_{sp}^{z}$ [40].

Considering the DDI between the components of the system within the near-field approximation, and using a Hamiltonian in terms of the one- and two-photon detunings, in the rotating wave approximation, we solve the Lindblad master equation for the density matrix elements to obtain [40]:

(1a) $$\begin{array}{cc} {\dot{\rho }}_{13}= & ‒\left[\left(\frac{\gamma_{13}}{2}+\frac{\gamma_{12}}{2}\right)+i\left(\Delta_{c}‒\Lambda_{z}\left(\rho_{33}‒\rho_{11}\right)\right)\right]\rho_{13}\\ \; & +i\Omega_{c}\left(\Pi_{z}+\Phi_{z}\right)\left(\rho_{33}‒\rho_{11}\right)+i\left[\Omega_{p}\left(\Pi_{x}+\Phi_{x}\right)+\Lambda_{x}\rho_{12}\right]\rho_{23}, \end{array}$$

(1b) $$\begin{array}{cc} {\dot{\rho }}_{12}= & ‒\left[\left(\frac{\gamma_{13}}{2}+\frac{\gamma_{12}}{2}\right)+i\left(\Delta_{p}‒\Lambda_{x}\left(\rho_{22}‒\rho_{11}\right)\right)\right]\rho_{12}\\ \; & +i\Omega_{p}\left(\Pi_{x}+\Phi_{x}\right)\left(\rho_{22}‒\rho_{11}\right)+i\left[\Omega_{c}\left(\Pi_{z}+\Phi_{z}\right)+\Lambda_{z}\rho_{13}\right]\rho_{32}, \end{array}$$

(1c) $$\begin{array}{cc} {\dot{\rho }}_{32}= & ‒\left(\frac{\gamma_{32}}{2}+i\Delta_{2}\right)\rho_{32}+i\left[\Omega_{c}^{*}\left(\Pi_{z}^{*}+\Phi_{z}^{*}\right)+\Lambda_{z}^{*}\rho_{31}\right]\rho_{12}\\ \; & ‒i\left[\Omega_{p}\left(\Pi_{x}+\Phi_{x}\right)+\Lambda_{x}\rho_{12}\right]\rho_{31}, \end{array}$$

(1d) $$\begin{array}{cc} {\dot{\rho }}_{11}= & ‒\left(\gamma_{12}+\gamma_{13}\right)\rho_{11}+i\left[\Omega_{c}\left(\Pi_{z}+\Phi_{z}\right)+\Lambda_{z}\rho_{13}\right]\rho_{31}\\ \; & +i\left[\Omega_{p}\left(\Pi_{x}+\Phi_{x}\right)+\Lambda_{x}\rho_{12}\right]\rho_{21}+c.c., \end{array}$$

(1e) $${\dot{\rho }}_{22}=\gamma_{12}\rho_{11}+\gamma_{32}\left(\rho_{33}‒\rho_{22}\right)‒i\left[\Omega_{p}\left(\Pi_{x}+\Phi_{x}\right)+\Lambda_{x}\rho_{12}\right]\rho_{21}+c.c.,$$

(1f) $${\dot{\rho }}_{33}=\gamma_{13}\rho_{11}+\gamma_{32}\left(\rho_{22}‒\rho_{33}\right)‒i\left[\Omega_{c}\left(\Pi_{z}+\Phi_{z}\right)+\Lambda_{z}\rho_{13}\right]\rho_{31}+c.c.,$$

In Equations (1), $\gamma_{1i}$ stand for the spontaneous decay rates of the QD excited level and $\gamma_{32}$ accounts for the lower states’ dephasing. $\Omega_{p}\left(\Pi_{x}+\Phi_{x}\right)$ represents the probe field Rabi frequency enhanced by the DDI for $|\Pi_{x}+\Phi_{x}|>1$ whereas $Im[\Lambda_{x}\left(\rho_{22}‒\rho_{11}\right)]$ gives the dipole contribution that enhances the total decay rate of the system. The dipole contribution from MNP and GND due to the probe field polarized along x direction and the control field polarized along z direction are given by $\Pi_{x,z}$ and $\Phi_{x,z}$ whereas $\Lambda_{x,z}$ arises when these fields polarize the QD which in turn polarizes MNP and GND. Π, Φ and Λ are defined for our system shown in Figure 1 by [40]:

(2a) $$\begin{array}{c} \Pi_{x}=\frac{1}{4\pi \epsilon^{*}}\left[\frac{\alpha_{G}^{x}\left(3cos\phi_{1}‒1\right)}{R_{QG}^{3}}+\frac{\alpha_{M}\left(3cos\phi_{2}‒1\right)}{R_{QM}^{3}}\right], \end{array}$$

(2b) $$\Phi_{x}=\frac{‒\alpha_{G}^{x}\alpha_{M}}{{\left(4\pi \epsilon^{*}\right)}^{2}R_{GM}^{3}}\left[\frac{3cos\phi_{1}‒1}{R_{QG}^{3}}+\frac{3cos\phi_{2}‒1}{R_{QM}^{3}}\right],$$

(2c) $$\Lambda_{x}=\frac{\mu_{12}^{2}}{{\left(4\pi \epsilon^{*}\right)}^{2}\hbar \epsilon_{0}\epsilon_{b}}\left[\frac{\alpha_{G}^{x}{\left(3cos\phi_{1}‒1\right)}^{2}}{R_{QG}^{6}}+\frac{\alpha_{M}{\left(3cos\phi_{2}‒1\right)}^{2}}{R_{QM}^{6}}\right],$$

(2d) $$\Pi_{z}=\frac{1}{4\pi \epsilon^{*}}\left[\frac{\alpha_{G}^{z}\left(3cos\theta_{G}‒1\right)}{R_{QG}^{3}}+\frac{\alpha_{M}\left(3cos\theta_{M}‒1\right)}{R_{QM}^{3}}\right],$$

(2e) $$\Phi_{z}=\frac{2\alpha_{G}^{z}\alpha_{M}}{{\left(4\pi \epsilon^{*}\right)}^{2}R_{GM}^{3}}\left[\frac{3cos\theta_{G}‒1}{R_{QG}^{3}}+\frac{3cos\theta_{M}‒1}{R_{QM}^{3}}\right],$$

(2f) $$\Lambda_{z}=\frac{\mu_{13}^{2}}{{\left(4\pi \epsilon^{*}\right)}^{2}\hbar \epsilon_{0}\epsilon_{b}}\left[\frac{\alpha_{G}^{z}{\left(3cos\theta_{G}‒1\right)}^{2}}{R_{QG}^{6}}+\frac{\alpha_{M}{\left(3cos\theta_{M}‒1\right)}^{2}}{R_{QM}^{6}}\right],$$

$\alpha_{G}^{x,z}$ and $\alpha_{M}$ are the shape dependent polarizabilities of GND and MNP respectively [42]. $\epsilon^{*}$ represents the effective dielectric constant of the system. Using iterative perturbation theory in the weak field limit for the probe field, one can write the coherence terms as [43]:

(3) $$\rho_{ij}=\rho_{ij}^{\left(0\right)}+\rho_{ij}^{\left(1\right)}+.......\rho_{ij}^{\left(n\right)}$$

The initial population is assumed to be in the ground state |2〉, thus:

(4) $$\rho_{11}^{\left(0\right)}=0,\;\rho_{22}^{\left(0\right)}=1,\;\rho_{33}^{\left(0\right)}=0.$$

By solving Equations (1b) and (1c) at steady state with $\rho_{31}^{\left(1\right)}=0$, we obtain the dynamics initiated by the probe field at first order:

(5) $$\rho_{12}^{\left(1\right)}=\frac{i\Omega_{p}\left(\Pi_{x}+\Phi_{x}\right)\left(\rho_{22}^{\left(0\right)}‒\rho_{11}^{\left(0\right)}\right)}{F}$$

where:

(6) $$F=\left(\frac{\gamma_{12}}{2}+\frac{\gamma_{13}}{2}\right)+i\left(\Delta_{p}‒\Lambda_{x}\right)+\frac{\Omega_{c}^{2}{\left|\Pi_{z}+\Phi_{z}\right|}^{2}}{\left(\frac{\gamma_{32}}{2}+i\Delta_{2}\right)}$$

Similarly, the dynamics initiated by the probe field at third order is:

(7) $$\rho_{12}^{\left(3\right)}=\frac{i\Omega_{p}\left(\Pi_{x}+\Phi_{x}\right)\left(\rho_{22}^{\left(2\right)}‒\rho_{11}^{\left(2\right)}\right)}{F}$$

With assuming $\rho_{33}^{\left(2\right)}=0$ and through the use of Equation (3), we get [44]:

(8) $$\rho_{22}^{\left(2\right)}‒\rho_{11}^{\left(2\right)}=\frac{‒4}{2\gamma +\gamma_{12}}\left[i\left(\Omega_{p}\left(\Pi_{x}+\Phi_{x}\right)+\Lambda_{x}\rho_{12}^{\left(1\right)}\right)\rho_{21}^{\left(1\right)}+c.c.\right]‒\frac{2\gamma_{32}}{2\gamma +\gamma_{12}}$$

where:

(9) $$\gamma =\frac{\gamma_{12}+\gamma_{13}+\gamma_{32}}{2}$$

Substituting from Equation (8) into Equation (7) gives:

(10) $$\begin{array}{cc} \rho_{12}^{\left(3\right)}= & \frac{i\Omega_{p}\left(\Pi_{x}+\Phi_{x}\right)}{F}\\ \; & \times \left[\frac{‒4\Omega_{p}^{2}{\left|\Pi_{x}+\Phi_{x}\right|}^{2}}{2\gamma +\gamma_{12}}\left(\frac{1}{F}+\frac{1}{F^{*}}+\frac{i(\Lambda_{x}‒\Lambda_{x}^{*})}{{\left|F\right|}^{2}}\right)‒\frac{2\gamma_{32}}{2\gamma +\gamma_{12}}\right] \end{array}$$

Thus, $\rho_{12}$ determined to third order is:

(11) $$\begin{array}{cc} \rho_{12}= & \frac{i\Omega_{p}\left(\Pi_{x}+\Phi_{x}\right)}{F}\\ \; & \times \left[\left(1‒\frac{2\gamma_{32}}{2\gamma +\gamma_{12}}\right)‒\frac{4\Omega_{p}^{2}{\left|\Pi_{x}+\Phi_{x}\right|}^{2}}{2\gamma +\gamma_{12}}\left(\frac{1}{F}+\frac{1}{F^{*}}+\frac{i(\Lambda_{x}‒\Lambda_{x}^{*})}{{\left|F\right|}^{2}}\right)\right] \end{array}$$

Therefore, the total susceptibility for the probe field is given by:

(12) $$\begin{array}{cc} \chi = & \frac{2iNd_{12}}{\epsilon_{0}E_{p}}\frac{\Omega_{p}\left(\Pi_{x}+\Phi_{x}\right)}{F}\\ \; & \times \left[\left(1‒\frac{2\gamma_{32}}{2\gamma +\gamma_{12}}\right)‒\frac{4\Omega_{p}^{2}{\left|\Pi_{x}+\Phi_{x}\right|}^{2}}{2\gamma +\gamma_{12}}\left(\frac{1}{F}+\frac{1}{F^{*}}+\frac{i(\Lambda_{x}‒\Lambda_{x}^{*})}{{\left|F\right|}^{2}}\right)\right] \end{array}$$

Comparing to the definition of the probe field susceptibility up to third order, $\chi =\chi^{\left(1\right)}+3\epsilon_{0}E_{p}^{2}\chi^{\left(3\right)}$[6], we can write the linear $\chi^{\left(1\right)}$ and the third-order nonlinear susceptibilities $\chi^{\left(3\right)}$ as:

(13a) $$\begin{array}{c} \chi^{\left(1\right)}=\frac{2iNd_{12}^{2}}{\epsilon_{0}\hbar }\frac{\left(\Pi_{x}+\Phi_{x}\right)}{F}\left(1‒\frac{2\gamma_{32}}{2\gamma +\gamma_{12}}\right) \end{array}$$

(13b) $$\chi^{\left(3\right)}=\frac{‒8iNd_{12}^{4}}{3\epsilon_{0}\hbar^{3}}\frac{\left(\Pi_{x}+\Phi_{x}\right){\left|\Pi_{x}+\Phi_{x}\right|}^{2}}{2\gamma +\gamma_{12}}\frac{1}{F}\left(\frac{1}{F}+\frac{1}{F^{*}}+\frac{i(\Lambda_{x}‒\Lambda_{x}^{*})}{{\left|F\right|}^{2}}\right)$$

It can be seen from Equation (13a), that the system can demonstrate amplification without population inversion, negative $Im[\chi^{\left(1\right)}]$, for $2\gamma_{32}<2\gamma +\gamma_{12}$, and negative real parts of $\left(\Pi_{x}+\Phi_{x}\right)$ that can be achieved with the present system in the optical region of the electromagnetic spectrum [40]. In terms of the linear and third-order nonlinear susceptibilities, the nonlinear Kerr index of refraction $n_{2}$ and nonlinear coefficient of absorption β for the probe field are given by [6]:

(14a) $$\begin{array}{c} n_{2}=\frac{3Re\left[\chi^{\left(3\right)}\right]}{4\epsilon_{0}cn_{0}^{2}}\;\mathrm{and}\;n_{0}=\sqrt{1+Re\left[\chi^{\left(1\right)}\right]} \end{array}$$

(14b) $$\beta =\frac{\omega }{\epsilon_{0}c^{2}n_{0}^{2}}Im\left[\chi^{\left(3\right)}\right]$$

$n_{0}$ in the above equations is the linear refractive index.

## 3. Analysis of Self-Kerr Nonlinearity

To study the self-Kerr nonlinearity in the MNP-GND-QD hybrid system and examine to what extent this type of nonlinearity can be controlled by the geometrical features of the system and the strength of the control field, we use the same parameters as in Ref. [40]. Consider GND of radius $L_{z}=7$ nm and thickness of $L_{x}=0.5$ nm at Fermi energy of 1.36 eV, temperature of 300 K and carriers’ mobility of $10^{4}\;{\mathrm{cm}}^{2}/\mathrm{Vs}$. With these parameters for GND embedded in GaAs background, the localized surface plasmon resonances are $\hbar \omega_{sp}^{x}=2.1724$ eV and $\hbar \omega_{sp}^{z}=0.6418$ eV [40]. We also consider a spherical silver nanoparticle of radius $R_{M}=15$ nm, $\epsilon_{\infty }=5.7$, $\omega_{p}=1.36\times 10^{16}\;s^{‒1}$ and damping rate for plasmons of $\gamma_{M}=10^{14}\;s^{‒1}$ [45].

The atomic parameters of CdSe self-assembled QD [46], chosen so as to support the strength of the DDI between the components of the system due to the relativity small dielectric constant of CdSe and its optical emission band [40], are set as $N=10^{20}\;m^{‒3}$, $\gamma_{12}=\gamma_{13}=2\pi \times 2$ GHz and $\gamma_{32}=0.3\gamma_{12}$ respectively [47]. The probe field applied along x direction induces surface plasmon polaritons in GND of energy $\hbar \omega_{sp}^{x}=2.1724$ eV that are resonant with excitons of CdSe self-assembled QD causing energy transfer through the coupling between them. The value of the control field Rabi frequency is chosen to match EIT conditions, i.e., $\Omega_{c}\geq \gamma_{12}$, and $|\Omega_{c}{|}^{2}>>\gamma_{12}\gamma_{32}$ [27].

**Figure 2:**
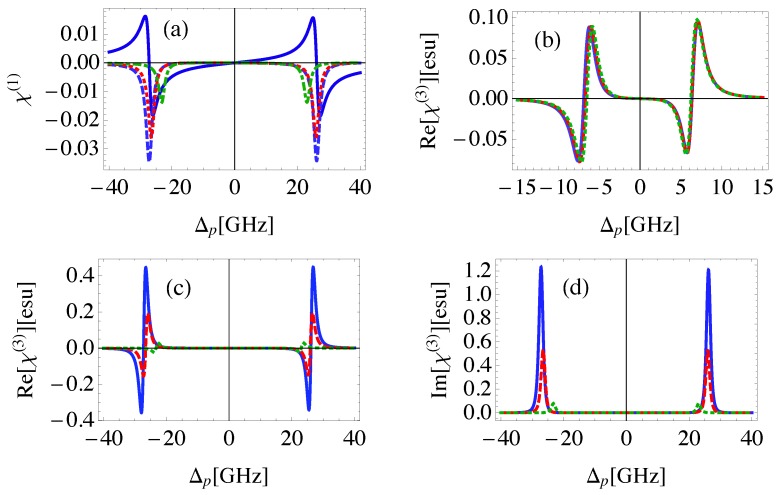
(**a**): The real (solid) and imaginary (dashed,dotted,dashed-dotted) parts of the linear susceptibility for the MNP-GND-QD hybrid system of $R_{GM}=23$ nm, $\theta_{G}=1$ rad, $\theta_{M}=0.4$ rad (solid,dashed), $\theta_{M}=0.45$ rad (dotted), $\theta_{M}=0.5$ rad (dashed-dotted). (**b**): The real part of the third order nonlinear susceptibility for the system of $R_{GM}=27$ nm, $\theta_{G}=1$ rad, $\theta_{M}=0.4$ rad (solid), $\theta_{M}=0.45\;\mathrm{rad}$ (dashed), $\theta_{M}=0.5$ rad (dotted). (**c**,**d**): The real (**c**) and imaginary (**d**) parts of the third order nonlinear susceptibility for the system of $R_{GM}=23$ nm, with the same values of $\theta_{G}$ and $\theta_{M}$ presented in (**b**). The system is excited with resonant control field of $\Omega_{c}=50$ GHz.

The linear and the third-order nonlinear susceptibilities are shown in Figure 2 for different geometrical parameters of the system defined in Figure 1. Note that as the inclination angle of MNP with respect to QD, $\theta_{M}$, decreases, $R_{QG}$ decreases. It can be seen from the linear susceptibility that the system demonstrates anomalous dispersion near resonance associated with an EIT window in the absorption spectrum as illustrated in Figure 2a. The splitting in the absorption spectrum is induced by the control field that generates dressed states between the atomic system (QD) and the plasmons of GND [48].

**Figure 3:**
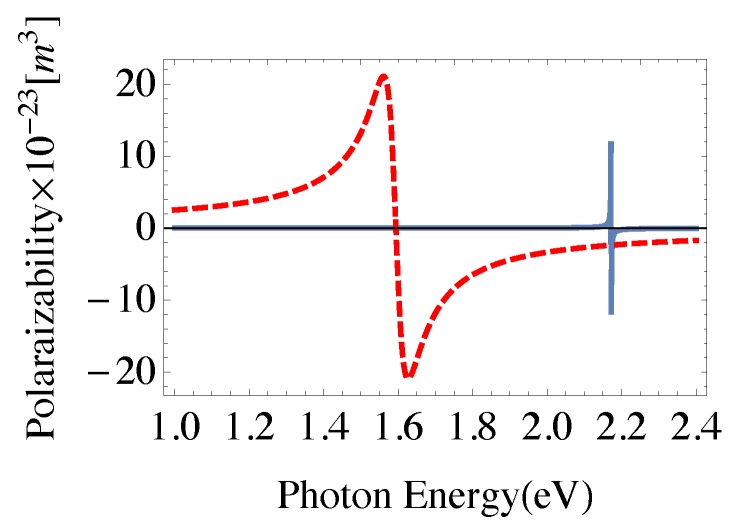
The real part of polarizability induced by x-polarized probe field for; (solid) GND of $L_{z}=7\;\mathrm{nm}$ and $L_{x}=0.5$ nm with Fermi energy of 1.36 eV, temperature of 300 K and carrier’s mobility of $10^{4}\;{\mathrm{cm}}^{2}/\mathrm{Vs}$, and for; (dashed) MNP of radius $R_{M}=15$ nm, $\epsilon_{\infty }=5.7$, $\omega_{p}=1.36\times 10^{16}\;s^{‒1}$ and damping of $\gamma_{M}=10^{14}\;s^{‒1}$. The plasmonic components are embedded in GaAs.

Amplification of the probe field without population inversion, due to the negative absorption coefficient is obtained at positions of maximum positive dispersion, and decreases as $\theta_{M}$ increases. This is due to the corresponding relatively large $R_{QG}$ that leads to relatively small local field enhancement resulting from the Coulomb interaction between GND and QD. The ability of the medium to amplify the probe field is an inherent property of the system arising from the negative real part of polarizability for both GND and MNP at the energy of the localized surface plasmon resonance of GND induced by the probe field as shown in Figure 3. Recently, the ability of plasmonic hybrid systems of demostrating gain without population inversion has been investigated in QD located in the vicinity of MNP [49,50]. It was shown that when such a system is exposed to a laser field and the distance between the quantum dot and the MNP is reduced beyond a critical value, a significant amount of gain without inversion is generated in the quantum dot [49]. The nonlinear optical properties of the system are enhanced for small $\theta_{M}$ as shown in Figure 2c,d due to the large local field enhancement for small $\theta_{M}$. The sensitivity of the nonlinear optical properties of the system to the geometrical parameters, $R_{QG}$ and $R_{QM}$, vanishes for large $R_{GM}$ as shown in Figure 2b emphasizing the important role of MNP in the self-Kerr nonlinearity of the system. It can be seen that the width of EIT window in the linear and nonlinear absorption spectra increases slightly as $\theta_{M}$ decreases.

**Figure 4:**
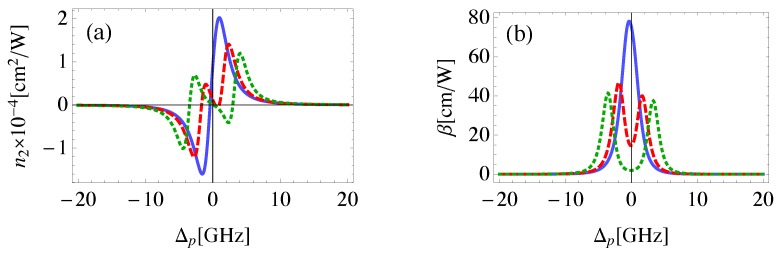
The self-Kerr nonlinear index of refraction (**a**) and nonlinear absorption coefficient (**b**) in the MNP-GND-QD hybrid system of $R_{QG}=12$ nm, $\theta_{G}=1$ rad, $\theta_{M}=0.5$ rad excited with resonant control field of $\Omega_{c}=1$ GHz. (solid), $\Omega_{c}=5$ GHz (dashed) and $\Omega_{c}=10$ GHz (dotted).

Although, EIT is typically associated with linear optical response, it can be also induced in the nonlinear response if the control field is strong enough [51]. This fact can be realized from Figure 4, where the EIT window is seen to emerge at relatively large values for the Rabi frequency of the control field. The self- Kerr nonlinear index of refraction obtained with Rabi frequency of 1GHz is qualitatively in agreement with that found experimentally by H. Wang et al. [31] with two orders of magnitude enhancement due to the present plasmonic system. The most important result is that the $\Omega_{c}$ required to demonstrate EIT in the nonlinear response is relatively small compared to $\gamma_{12}$ due to the large local field enhancement induced by the plasmonic components of the system at small center-to-center distances between them, resulting in more effective excitation. It can be seen that a giant self-Kerr nonlinearity is obtained which is enhanced by several orders of magnitude compared to those of traditional nonlinear materials and MNP-QD hybrid systems [6,52].

**Figure 5:**
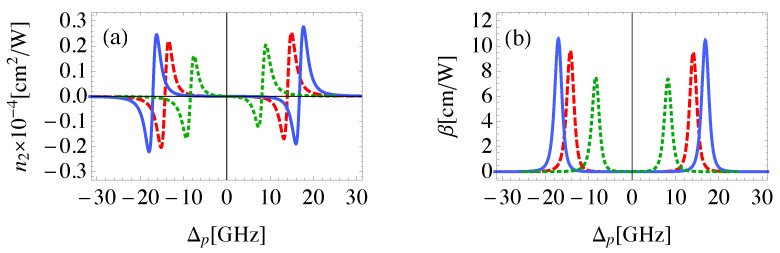
The self-Kerr nonlinear index of refraction (**a**) and nonlinear absorption coefficient (**b**) in the MNP-GND-QD hybrid system of $R_{QG}=13$ nm, $\theta_{G}=1$ rad, $R_{GM}=26\;\mathrm{nm},\theta_{M}=0.55$ rad (solid), $R_{GM}=28\;\mathrm{nm},\theta_{M}=0.5$ rad (dashed) and $R_{GM}=34\;\mathrm{nm},\theta_{M}=0.4$ rad (dotted). The system is excited with resonant control field of $\Omega_{c}=50$ GHz.

Since the strength of the DDI between the components of the system is relatively large for small distances between GND and MNP [40], we examine the possibility to control the Kerr nonlinearity of the system by using $R_{GM}$ at constant $R_{QG}$. Figure 5 shows the dependence of the self-Kerr nonlinear properties on the $R_{GM}$. We observe that $n_{2}$ and β increase, with red shifted (blue shifted) resonances for positive (negative) probe field detuning as $R_{GM}$ decreases. It is clear that the width of the EIT window is sensitive to the distances between GND and MNP. Moreover, switching between positive and negative $n_{2}$ is observed and can be controlled via the probe field detuning as well as the geometrical parameters of the system. This switching is useful to manipulate the wave front of propagating wave through self-focusing that induces large light intensities, and in turn to protect the material from damaging via self-defocusing.

**Figure 6:**
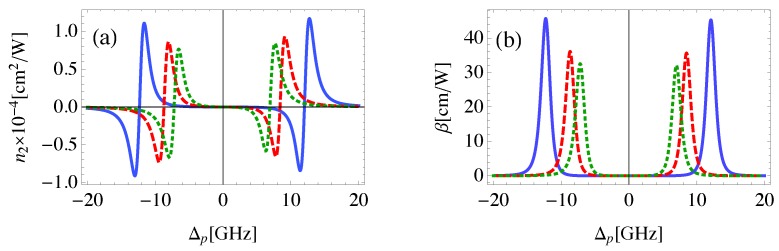
The self-Kerr nonlinear index of refraction (**a**) and nonlinear absorption coefficient (**b**) in the MNP-GND-QD hybrid system of $R_{QG}=12$ nm, $R_{GM}=26$ nm, $\theta_{M}=0.5$ rad, $\theta_{G}=1$ rad, $R_{M}\;=18$ nm (solid), $R_{M}=16$ nm (dashed) and $R_{M}=15$ nm (dotted), The system is excited with resonant control field of $\Omega_{c}=50$ GHz.

It was found that the rate of energy exchange between plasmons and excitons in the MNP-GND-QD hybrid system depends crucially on the size of MNP giving a sense of the role of MNP in the nonlinearity of the system [40]. Therefore, the effect of the MNP size on the self-Kerr nonlinearity of the system is investigated in Figure 6. It can be seen that large self-Kerr nonlinear index of refraction and coefficient of absorption are obtained with relatively large size of MNP. The resonances of nonlinear optical response are red shifted (blue shifted) for positive (negative) probe field detuning as $R_{M}$ increases, leading to a relatively wide EIT window. Apparently, the magnitude of the self-Kerr nonlinear optical properties of the system is sensitive to the size of MNP.

It is worth mentioning here that the shifting of resonances is obvious when we change the size of MNP and $R_{GM}$ and is more significant than that obtained by changing $\theta_{M}$ because of the strong dependence of dipole contributions ($\Pi_{x}$,$\Phi_{x}$ and $\Lambda_{x}$) on the two former parameters. Specifically, the shifting of resonances becomes evident by noting the term $\Delta_{p}‒\Lambda_{x}$ in Equation (6). Moreover, in typical linear response EIT, the two resonances are symmetric around zero probe field detuning. On the other hand, the nonlinear response depends on the two-photon detuning which is affected by the sign of the probe field detuning. The asymmetry in the lineshape becomes obvious as the nonlinear absorption decreases for small MNP size and relatively large center-to-center distances between components of the system. This is reasonable since the latter cases are associated with relatively small $\Pi_{x}$, $\Phi_{x}$ and $\Lambda_{x}$ (note Equations (6) and (13b)).

**Figure 7:**
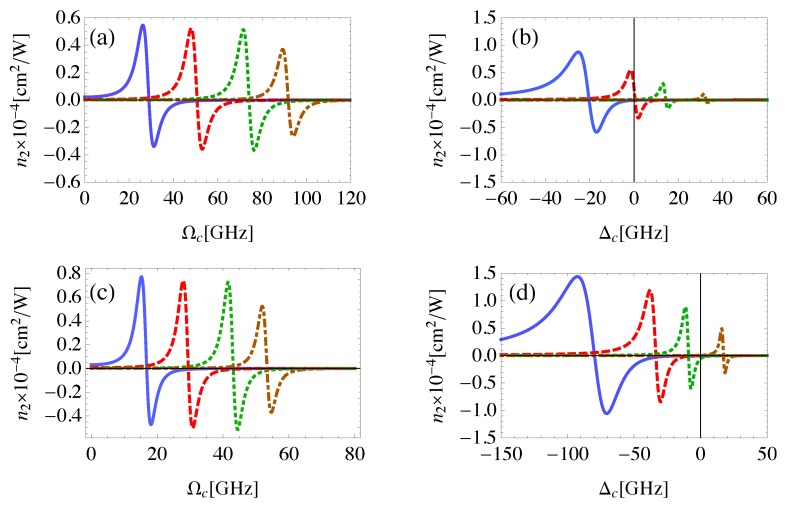
The self-Kerr nonlinear index of refraction for the probe field in the MNP-GND-QD hybrid system of $R_{M}=15$ nm (**a**,**b**), $R_{M}=18$ nm (**c**,**d**), $R_{GM}=26$ nm, $\theta_{M}=0.5$ rad, $\theta_{G}=1$ rad, excited with control field of (**a**,**c**): $\Delta_{c}=0$, (**b**,**d**): $\Omega_{c}=50$ GHz and probe field of detuning $\Delta_{p}=10$ GHz (solid), $\Delta_{p}=18$ GHz (dashed), $\Delta_{p}=26$ GHz (dotted), and $\Delta_{p}=40$ GHz (dashed-dotted).

In order to study the influence of the control field Rabi frequency on the self-Kerr nonlinearity, we consider the case when the control field is at resonance, with different values of probe field detuning as shown in Figure 7a,c. We observe that the self-Kerr nonlinear index of refraction decreases as the detuning of the probe field increases. For each value of probe field detuning there is a value of control field Rabi frequency that maximizes $n_{2}$, and increases as $\Delta_{p}$ increases. We find that the sign of $n_{2}$ can be controlled by the $\Omega_{c}$, in addition to $\Delta_{p}$, achieving a switching between self-focusing and self-defocusing. Figure 7b,d shows the dependence of the self-Kerr nonlinearity on the detuning of the control field at different values of the probe field detuning. The most important remark is that, large self-Kerr nonlinearity is induced with small $\Delta_{p}$ and large negative $\Delta_{c}$ due to the large corresponding two-photon detuning that enhances the self-Kerr nonlinearity of the system as noted by Equations (6) and (13b). The self-Kerr nonlinearity vanishes for small values of two-photon detuning.

Moreover, the interplay between the MNP size, the Rabi frequency and the detuning of the control field in order to maximize the self-Kerr nonlinearity is investigated in Figure 7. The externally controlled Rabi frequency and detuning of the control field are adjusted to maximize the self-Kerr nonlinearity for different sizes of MNP. We observe inversely proportional relation between the size of the MNP and the control field Rabi frequency required to maximize the self-Kerr nonlinearity as shown in Figure 7a,c. This is reasonable since the strength of the DDI between the components of the system is enhanced for relatively large size of MNP within the limits of the near-field approximation. On the other hand, $\Delta_{c}$ required to maximize the self-Kerr nonlinearity takes larger negative values with larger size of MNP to compensate for the relatively small $|\Pi_{z}+\Phi_{z}|$ in such a case (note Equation (6)).

**Figure 8:**
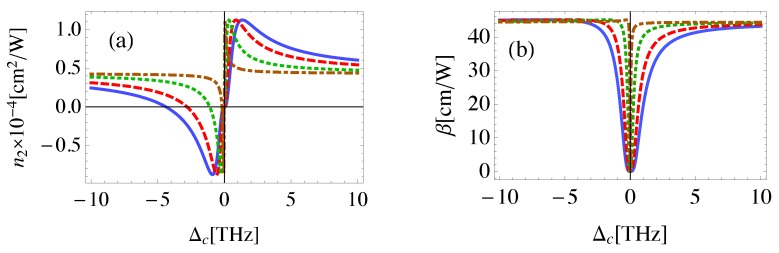
The self-Kerr nonlinear index of refraction (**a**) and nonlinear absorption coefficient (**b**) in the MNP-GND-QD hybrid system of $R_{GM}=26$ nm, $\theta_{M}=0.5$ rad, $\theta_{G}=1$ rad, $R_{M}=15$ nm excited with resonant probe field and control field of $\Omega_{c}=100$ GHz (solid), $\Omega_{c}=80$ GHz (dashed), $\Omega_{c}=50$ GHz (dotted) and $\Omega_{c}=20$ GHz (dashed-dotted).

Figure 8 shows that a relatively small control field Rabi frequency is required to achieve large $n_{2}$ at resonance for both probe and control fields. On the other hand, relatively small β is obtained at resonance showing a good agreement with the relevant experimental results. Specifically, using z-scan method, the nonlinear optical response in hybrid structures composed of CdTe QDs and periodic arrays of gold nanoparticles excited by 50 fs laser pulses of 800 nm wavelength has been studied. It was found that the largest nonlinear refractive index, i.e., $-0.53\;{\mathrm{cm}}^{2}/\mathrm{GW}$ and the smallest nonlinear absorption coefficient, i.e., 25 cm/GW are obtained when the Au surface plasmon polaritons are resonant with excitons in the CdTe QDs [52]. Our results are also in agreement with a recent experimental study that demonstrated significant enhancement in the nonlinear optical properties of ZnO nanoparticles near graphene nanosheets [53].

**Figure 9:**
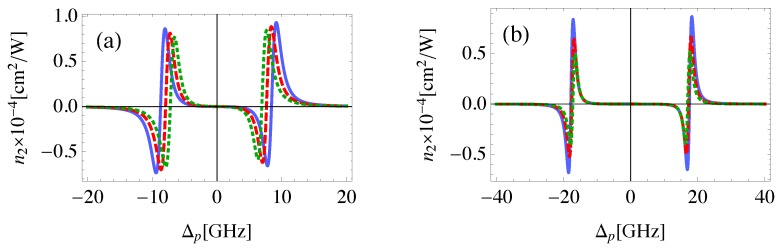
The self-Kerr nonlinear index of refraction for the probe field in the MNP-GND-QD hybrid system of $R_{QG}=12$ nm, $R_{GM}=26$ nm, $\theta_{G}=1$ rad, (**a**): $\theta_{M}=0.5$ rad, $R_{M}=16$ nm (solid), $R_{M}\;=\;15.5$ nm (dashed) and $R_{M}=15$ nm (dotted), (**b**): $R_{M}=15$ nm, $\theta_{M}=0.5$ rad (solid), $\theta_{M}=0.52$ rad (dashed) and $\theta_{M}=0.54$ rad (dotted). The system is excited with resonant control field of $\Omega_{c}=50$ GHz.

Since the robustness of the nonlinear system is a fundamental requirement for its practical value in real-world applications, we finally examine the nonlinear optical properties of the system under small variations of its parameters; namely, the geometry of the system and the size of MNP as shown in Figure 9. It can be seen that the self-Kerr nonlinearity undergoes weakly monotonic change as a result of small variations of the parameter of the system. Nevertheless, the strong nonlinearity persists despite the parameter variation. Interestingly, the strength of the control field used in our analysis to induce the giant nonlinearity in the QD is about $3\times 10^{6}$ V/m. This value of the field strength is significantly smaller than the characteristic field associated with nonlinear effects in atoms [6], and one order of magnitude smaller than the electric field strength required to observe nonlinear effects in graphene of typical doping levels [54]. Thus, MNP-GND-QD hybrid systems can provide a promising platform for high nonlinearity below the laser induced damage threshold for the components of the system.

## 4. Conclusions

We have studied the self-Kerr nonlinearity in a MNP-GND-QD hybrid system in the optical region with CdSe self-assembled QD modeled as a three- level system of Λ configuration interacting with weak probe field and strong control field. We have derived analytical expressions for linear and third-order nonlinear susceptibilities for the probe field by solving the density matrix equations of the system at steady state in the weak probe field limit. A giant self-Kerr nonlinear index of refraction is obtained at resonance for the probe and control fields with relatively low light intensities. The linear optical response of the system demonstrates an EIT window and amplification without population inversion. The self-Kerr nonlinearity of the system is enhanced for small center-to-center distances between the components of the hybrid system and relatively large size MNP within the near-field limit. The Rabi frequency of the control field required to maximize the self-Kerr nonlinearity decreases as the detuning of probe field decreases and the size of MNP increases . The self-Kerr nonlinearity of the system vanishes for small values of the two-photon detuning. Our results exhibit robustness under small variation of the system’s parameters implying its potential for practical applications.

Such giant and controllable self-Kerr nonlinearity could be employed in interesting applications of optoelectronics devices such as all-optical switching. Moreover, the amplification without population inversion demonstrated by the system at relatively low light intensity could be used to construct efficient and controllable plasmonic amplifiers.

Although plasmonic structures can greatly enhance nonlinear light-matter interaction through the strong field enhancement, achieving high performance nonlinear plasmonic devices is limited by optical loss and material damage. With EIT we overcome the latter limitation since large nonlinearity is obtained with relatively low light intensity. Moreover, the former limitation is suppressed through using CdSe self-assembled QDs of small dielectric constant as gain medium incorporated within the hybrid plasmonic system of highly doped GNDs and relatively large size of MNPs that induces large local field enhancement for small center-to-center distances between the components of the system.

We hope that this work will stimulate further theoretical and experimental investigations which will contribute to a better understanding of the nonlinearity of MNP-GND-QD hybrid nanostructures and their potential applications.
